# Beyond spectral moments: Validating alternative measures of sibilant fricatives using listener ratings of children's speech

**DOI:** 10.1121/10.0039497

**Published:** 2025-10-01

**Authors:** Eugene Wong, Benjamin Munson

**Affiliations:** Department of Speech-Language-Hearing Sciences, University of Minnesota, Minneapolis, Minnesota 55455, USA wong0703@umn.edu, munso005@umn.edu

## Abstract

Shadle [(2023). J. Acoust. Soc. Am. **153**, 1412–1426] proposed that the spectral peak in mid-frequency (*F_M_*) is a superior measure of place of articulation of sibilant fricatives to the most commonly used measure, the first spectral moment (M1). It is examined as to whether *F_M_* predicts adult listener's ratings of the place of articulation of 2.5–3.5-year-old children's word-initial /s/ and /ʃ/ when compared to M1. Regression models reveal that *F_M_* in 3–9 kHz range best predicts listener's ratings of children's fricatives. These results provide additional validation for *F_M_* as a measure of fricatives' place of articulation, including in children's speech.

## 1. Introduction

The acquisition of English /s/ and /ʃ/ is protracted. Studies using impressionistic phonetic transcription show that typically developing, English-speaking 6–9-year-old children in the Midwestern United States still produce about 10% of target /s/ and /ʃ/ sounds in error (e.g., Smit, 1993). Producing fricatives is inherently articulatorily challenging as they require a constriction that is narrow enough to cause turbulent airflow but not so narrow that it stops the airflow altogether (Kent, 1992). Given this, for some children, the production of /s/ and /ʃ/ may be particularly difficult, leading to productions that are transcribed as errors. This is especially common in children with speech sound disorder (SSD). Children with SSD may, for example, delete or substitute a stop sound for either fricative. Place of articulation errors also occur. For the target /ʃ/, children with SSD often produce a sound that is transcribed as [s], or a sound whose place of articulation is intermediate between [s] and [ʃ]. For the target /s/, they may substitute it with a fronted sound, transcribed as [s̪] or [θ].

Acoustic analyses of children's speech have indicated that children's fricative errors are often not true substitutions. For example, Baum and McNutt (1990) showed that children's productions of [θ] for /s/ and [θ] for /θ/ were acoustically distinct. Similar results have been found for [s] for /ʃ/ errors, which are distinct from correct productions of /s/ (Li *et al.*, 2009). Those findings are consistent with a model of speech sound development as a process of gradual differentiation of sounds from one another (Li, 2012; Li and Munson, 2016; Romeo *et al.*, 2013), beyond the point at which adults transcribe children's productions as correct. In short, acoustic measurement of fricatives is an important tool for understanding speech sound development. It provides a highly detailed, fine-grained measurement of speech production accuracy. That highly granular nature means that it is useful for monitoring intervention progress in cases where children receive speech therapy of /s/ and /ʃ/ misarticulation (Munson *et al.*, 2012).

The research described in the previous paragraph invites the question of how to best summarize the acoustic characteristics of fricatives. One frequently used analysis involves calculating spectral moments. Spectral moments treat the power spectrum of a fricative as a random distribution, and then calculate the mean, standard deviation (SD), skewness, and kurtosis (the first through fourth spectral moments [M1–M4]) of this distribution (Forrest *et al.*, 1988). Numerous empirical studies support the use of spectral moments. M1, also known as spectral centroid, spectral mean, or center of gravity, is widely used to characterize sibilant fricatives in the literature. M1 differentiates /s/ from /ʃ/ with high accuracy (Jongman *et al.*, 2000). M1 was used as the primary outcome measure in the studies by Li (2012) and Romeo *et al.* (2013), cited earlier. Moreover, a host of sociophonetic studies on /s/ shows that variation in M1 is socially meaningful as it is related to attributes such as actual and perceived gender in adults' and children's speech (e.g., Houle *et al.*, 2023; Munson *et al.*, 2015; Stuart-Smith, 2007; Wong *et al.*, 2025). Given its utility, it is not surprising that spectral moments are used widely and are even incorporated directly into one of the most broadly used acoustic analysis systems in the world, Praat (Boersma and Weenink, 2023).

Despite the findings in the previous paragraph, spectral moments have several seemingly intractable flaws. These critiques have been described in great detail by Shadle (2023). One theme from these critiques is that spectral moments do not illuminate the articulatory-acoustic relationship for fricatives. Shadle summarized a set of alternative measures to spectral moments to characterize fricatives. Shadle *et al.* (2023) and Koenig *et al.* (2013) validated these measures by showing how well they are predicted by models of articulatory-acoustic relationships for fricatives and actual articulatory and acoustic data. These include measures that examine the acoustic characteristics of the noise source separate from the acoustics of the place of articulation. This study focuses on the latter.

Based on aerodynamic and articulatory data, studies from Shadle (1985), Shadle *et al.* (2016), Shadle *et al.* (2023), and Koenig *et al.* (2013), *inter alia* proposed using the spectral peak in mid-frequency range (*F_M_*, typically defined in these studies as the highest amplitude in the 3–7 kHz range) as a measure of resonance of front cavities during fricative production, from which inferences to the place of articulation or location of constriction can be made. Shadle *et al.* (2016) examined *F_M_* of adult /s/ tokens in the XRMB corpus (Westbury, 1994), which included articulatory and acoustic data. Jaw height was measured, and the length of front cavity was estimated based on the lip pellets and incisors across nine time-points during /s/ production. Results showed that *F_M_* corresponded to changes of length of front cavity across time-points, whereas M1 did not. This supported the use of *F_M_* as a measure of place of articulation.

Koenig *et al.* (2013) compared measures of *F_M_* and M1 using data of /s/ in single-word productions and connected speech from 12 typically developing adolescents of 10–15 years of age. Results showed that *F_M_* reflects the temporal changes in location of constriction more closely than M1. Another study by Shadle *et al.* (2023) examined fricative acoustics in connected speech. For *F_M_* measurement, the authors defined mid-frequency as 3–7 kHz for male speakers and 3–8 kHz for female speakers. Based on observations of spectra, Shadle *et al.* argued that increasing the upper limit of mid-frequency to 8 kHz for female speakers should yield more accurate results, but further extending the upper limit to 9 kHz was not recommended because the highest-amplitude peak occurring in such a range is often above the lowest resonating frequency related to the front cavity.

Work from Shadle and colleagues provided empirical support for *F_M_* in mid-frequency as an acoustic index of place of articulation in sibilant fricatives. The current paper advances this work in two ways. The first way is that it applies the measure of *F_M_* to the speech of young children, whose /s/ and /ʃ/ sounds are developing. This is important methodologically as it is unclear what frequency range is optimal for measuring *F_M_* in the speech of young children, whose fricatives have an overall distribution of energy in higher frequencies than those for adults (Munson, 2004). The first goal of the current study is descriptive: to examine the *F_M_* measure in the speech of young children and compare it to M1 for the same sounds.

A second goal of this investigation is to validate the *F_M_* measure by examining how well it predicts listeners' visual analog scale (VAS) ratings of the goodness of children's /s/ and /ʃ/ productions and contrasting that with how well M1 measures predict the same set of ratings. A finding that *F_M_* predicts ratings better than M1 would be further evidence of the superiority of this measure. There is much literature examining the utility of continuous ratings of children's speech. These were first introduced into the literature on speech development by Munson *et al.* (2010), who devised a task based on the demonstration of the utility of continuous ratings in the perception of adults' speech by Massaro and Cohen (1983). One representative study of VAS ratings of children's speech is Schellinger *et al.* (2017), who presented word-initial consonant-vowel (CV) sequences excised from real-word productions of target words beginning with /s/ or /θ/. Their stimulus set included tokens that had been transcribed as [s], [θ], or a sound intermediate between them. Munson and Urberg Carlson (2016) examined ratings of tokens of word-initial CVs excised from words beginning with target /s/ or /ʃ/ and transcribed as [s], [ʃ] or a sound intermediate between them. In both of those studies, VAS ratings correlated with the acoustic characteristics of the fricatives, such as the first (for [s] vs [ʃ]) and the second spectral moments (for [s] vs [θ]). The authors of these studies concluded that continuous ratings could serve as a proxy for acoustic measures in the assessment of children's speech. In the current study, VAS ratings serve as the “standard” against which the two acoustic measures, M1 and *F_M_*, are compared. We hypothesized that *F_M_* would be more strongly related to VAS ratings than M1, given that *F_M_* is a better predictor of articulatory location than M1.

## 2. Methods

### 2.1 Children's speech data

Sixty-three children participated in a longitudinal study of speech and vocabulary growth, the full results of which are summarized elsewhere (e.g., Erskine *et al.*, 2020). Speech data for the current study comes from a real-word repetition task in the first time-point of the study when children were 2.5–3.5 years of age (*M* = 2 years, 8 months; SD = 3.6 months). There were 31 male and 32 female children. These children were a random subset of the larger group of children who participated in the longitudinal study.

In the real-word repetition task, each child repeated approximately 100 English words after hearing an auditory prompt and seeing a picture on a computer. Children's responses were recorded using a Marantz portable recorder (Carlsbad, CA) with a sampling rate of 44.1 kHz. The test words include multiple English consonants and vowels. The /s/-initial words were *sick*, *scissors*, *soup*, *soap*, *sun*, *sad*, *sandwich*, and *sock*. The /ʃ/-initial words were *sheep*, *share*, *show*, *shower*, and *shovel*. Some test words were repeated more than once, where the goal was to elicit 32 productions of each target fricative, occurring equally before high and low vowels and front and back vowels.

Trained research assistants annotated the recordings in Praat (Boersma and Weenink, 2023) using an iterative process described in detail in Munson *et al.* (2021). The onset of fricative was visually identified based on the rise of turbulent noise in the waveform, whereas the offset of the fricative is defined as the offset of the turbulent noise or the beginning of periodic waveform of the following vowel. The productions analyzed in this study were selected because they had been transcribed as a sibilant fricative rather than a stop, an approximant, or a deletion. This allowed us to examine only those measures that reflect place of articulation and not, for example, characteristics of the noise source. All productions were narrowly transcribed as [s], [ʃ], [s]:[ʃ] (an intermediate sound that is perceived to be close to [s]), or [ʃ]:[s] (an intermediate sound that is perceived to be close to [ʃ]) based on the research assistant's auditory perception.

The acoustic properties of these fricatives were measured in Praat (Boersma and Weenink, 2023). First, each fricative was bandpass filtered at 300 Hz to remove any coarticulatory effects of voicing and low-frequency ambient noise. From the 80% duration centered at the temporal midpoint of the fricative, time-averaged spectra were obtained using six windows of 15 ms. Spectral centroid (M1) and the spectral peak (*F_M_*) in 3–7 kHz, 3–8 kHz, and 3–9 kHz ranges were extracted with a custom-written Praat script. M1 was measured as the weighted average of the frequencies in the amplitude normalized spectrum, whereas *F_M_* was measured as the frequency with the highest amplitude in the predetermined mid-frequency range. Pre-emphasis was not applied. We compared the results of spectral peak measurement from three mid-frequency ranges as it is unclear which frequency range best captures the lowest resonant frequency of fricatives in children of this age range in an automated procedure. Although not recommended by Shadle *et al.* (2023), the upper limit of 9 kHz was selected because young children often produce speech with higher frequencies than adolescents and adults.

Some previous work on children's production of /s/ and /ʃ/ has also examined the frequency of the second formant (F2) at vowel onset, which is needed to differentiate /s/ from /ʃ/ in some languages such as Japanese (Li *et al.*, 2009). It was not measured in the current study because Li (2012) found it not necessary to differentiate between /s/ and /ʃ/ in English-speaking children of the age range of the current study.

### 2.2 Adult listener's VAS ratings

A total of 76 adult naive listeners participated in a perception study. All of them were native speakers of English; 18–50 years of age with no past or present speech, language, or hearing disorders; and were recruited from the University of Minnesota community via flyers and word of mouth.

The stimuli were children's production of word-initial /s/ and /ʃ/ tokens and 150 ms of the following vowels. Listeners were asked to rate the accuracy of the fricative sound by clicking on a VAS of a double-sided arrow anchored with the text *The “s” sound* and *The “sh” sound* on the two ends. Listeners' click locations were logged. For the perception study, 1490 productions across 63 children were selected as stimuli because these productions were transcribed as a fricative and not produced with whispery voice or overlapped with background noises. These stimuli were divided into four blocks, where each listener rated approximately 536–576 stimuli. A total of 42 454 VAS ratings were elicited.

### 2.3 Data analysis

All VAS ratings of children's /s/ and /ʃ/ accuracy were transformed from the raw click location in pixels to a value ranging from zero (the tip of the arrow at the /s/ end) to one (the tip of the arrow at the /ʃ/ end). For the acoustic measurement of children's fricatives, we excluded tokens with total duration that were shorter than 60 ms, and outliers that were ±2 SD from the mean of each acoustic measure based on the target sound. As a result, 1193 tokens of children's fricatives and 33 652 ratings remained in the analysis. The distribution of tokens across four narrow transcription categories are shown in Table 1. As suggested by one of the anonymous reviewers, the maximum overlap of windows should not be more than 50%. Here, only eight tokens exceeded this criteria.

**Table 1.** Distribution of /s/ and /ʃ/ tokens across the four transcription categories.

Transcription	Target /s/	Target /ʃ/
[ʃ]	70	340
[ʃ]:[s]	18	79
[s]:[ʃ]	44	61
[s]	451	130
Total	583	610

Mixed-effect regression models with beta distribution were fitted for each acoustic measure using the glmmTMB package (Brooks *et al.*, 2017). Beta distribution was used to accommodate for the response variable, VAS ratings, which are bounded between zero and one. To facilitate comparisons, all acoustic measures were also scaled into zero to one. For each regression model, the fixed effects were the target (binary coded as /s/ = 1 and /ʃ/ = 0) and a single acoustic measure (i.e., M1, *F_M_* in 3–7 kHz, *F_M_* in 3–8 kHz, or *F_M_* in 3–9 kHz ranges). The baseline model was fitted as VAS ratings predicted by target. Main effects and interaction effects of target and the acoustic measures were examined through comparing model fit based on chi-square tests. The random effects were target and acoustic measure by adult listeners and intercept by stimuli. To determine the model fit across the models with different acoustic measures, the Akaike information criterion (AIC) values from the glmmTMB model output were compared.

## 3. Results and discussion

Table 2 shows the descriptive statistics of listener's VAS rating, M1, *F_M_* in 3–7 kHz, 3–8 kHz, and 3–9 kHz ranges across the four transcription categories. Overall, the mean values for all four acoustic measures reflected the differences in VAS ratings. For the three *F_M_* measures, the mean values increased as the upper limit of the mid-frequency range increased. The changes in mean values across the three *F_M_* ranges were smaller for the [ʃ] and [ʃ]:[s] than for [s] and [s]:[ʃ].

**Table 2.** Means (and SDs) of VAS ratings, spectral centroid (M1), spectral peak (*F_M_*) in 3–7 kHz, 3–8 kHz, and 3–9 kHz across four transcription categories.

Transcription	*N*	VAS rating	M1 (in Hz)	*F_M_* in 3–7 kHz	*F_M_* in 3–8 kHz	*F_M_* in 3–9 kHz
[ʃ]	410	0.75	4912.89	4255.65	4485.55	4557.86
		(0.20)	(1182.59)	(913.84)	(1236.63)	(1351.03)
[ʃ]:[s]	97	0.62	5257.71	4677.91	4915.53	4972.53
		(0.24)	(1291.43)	(1017.52)	(1341.76)	(1414.22)
[s]:[ʃ]	105	0.49	6242.00	5209.71	5880.32	6117.60
		(0.28)	(1489.07)	(1143.72)	(1452.99)	(1592.36)
[s]	581	0.29	6975.52	5640.43	6402.79	6769.01
		(0.23)	(1687.17)	(1125.68)	(1333.19)	(1470.80)

**Fig. 1.**
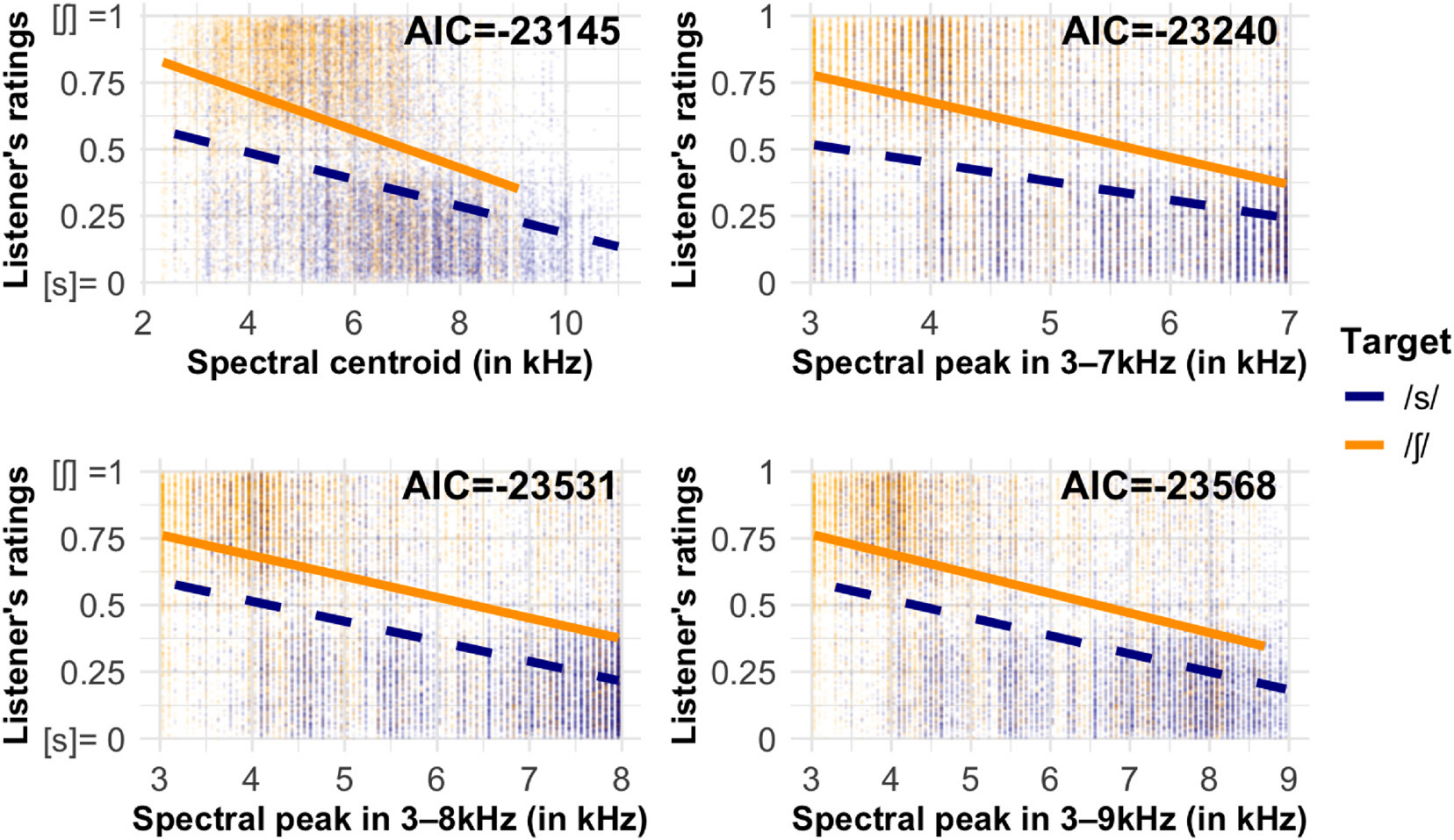
Linear relationships are noted between listener's VAS ratings of /s/ (dashed blue lines) and /ʃ/ (solid orange lines) accuracy and the acoustic measures of spectral centroid (m1; top left panel), spectral peak in 3–7 kHz range (top right panel), spectral peak in 3–8 kHz range (bottom left panel), and spectral peak in 3–9 kHz range (bottom right panel). AIC values for these models are shown in the top right corner within each panel.

Regression analysis showed that for M1, the model with the interaction effect of target*M1 had a significantly better fit than the model without the interaction effect (χ^2^_[df=6]_ = 1355.2, *p* < 0.001). Full model statistics can be found in the supplementary material. The relationship between M1 and VAS ratings for /s/ and /ʃ/ targets are displayed in the top left panel of Fig. 1. As can be observed from Fig. 1, the slope is higher for /ʃ/ than it is for /s/. This interaction effect may simply reflect the differences in the acquisition rates of /s/ and /ʃ/, and [s] was substituted for many /ʃ/ targets for these children. Another possible explanation is that M1 is less informative for judging /s/ than /ʃ/. One could consider that the sound close to /ʃ/ is /s/, which differs only in M1, whereas /s/ could be similar to /ʃ/ and /θ/, which may differ in M1 (when compared to /ʃ/) and M2 (the degree of diffusion in the spectrum when compared to /θ/). This explanation is consistent with that of Schellinger *et al.* (2017), who compared /ʃ/ and /θ/. Hence, the effect of M1 on VAS ratings may be stronger for /ʃ/ than /s/.

For spectral peak (*F_M_*) measurements, the models with main effects of target and *F_M_* at all three mid-frequency ranges showed significantly better fit than the baseline model (*F_M_* in 3–7 kHz, χ^2^_[df=7]_ = 819.12; *F_M_* in 3–8 kHz, χ^2^_[df=7]_ = 1109.5; *F_M_* in 3–9 kHz, χ^2^_[df=7]_ = 1146.6; all *p*'s < 0.001). There were no interaction effects. *F_M_* in all three mid-frequency ranges significantly predicted VAS ratings (*F_M_* in 3–7 kHz, *b*= −1.50; *F_M_* in 3–8 kHz, *b*= −1.61; *F_M_* in 3–9 kHz, *b*= −1.74, *p* < 0.001). The relationship between *F_M_* in each of the three mid-frequency ranges and VAS ratings for /s/ and /ʃ/ are depicted in the top right, bottom left, and bottom right panels of Fig. 1.

The model fits for these four models of M1 and *F_M_* predicting VAS ratings of children's /s/ and /ʃ/ accuracy were assessed by comparing their AIC values (displayed in the top right corner of each panel in Fig. 1). A smaller AIC value suggests better model fit. As shown in Fig. 1, models of *F_M_* in all three mid-frequency ranges showed lower AIC than M1. The model using *F_M_* in 3–9 kHz to predict listener ratings had the lowest AIC values, indicating best model fit among the four models. This also suggests that *F_M_* in 3–9 kHz was the best measure to reflect VAS ratings, and it was the most appropriate mid-frequency range to measure *F_M_* for the /s/ and /ʃ/ of 3-year-old children when compared to 3–7 kHz and 3–8 kHz ranges. Moreover, when compared to the model of *F_M_* in 3–8 kHz, the difference in AIC values of these two models was relatively small. This suggests that choosing an even higher upper limit of mid-frequency (e.g., 10 kHz) for *F_M_* would not likely improve model fit and may even result in erroneous measures that do not reflect the lowest resonating frequency of the front cavity. It should be noted that approximately 5% of the tokens showed a “substantial jump” in *F_M_* measurement. These tokens had a spectral peak less than 7 kHz when using 3–8 kHz as mid-frequency range but were above 8 kHz when using 3–9 kHz as mid-frequency range. Among these tokens, most were [s] tokens, but seven were transcribed as [ʃ], one was transcribed as [ʃ]:[s], eight were transcribed as [s]:[ʃ], suggesting possible measurement errors and supporting the conclusion from Shadle *et al.* (2023) that the 3–9 kHz range can include peaks other than the lowest resonances.

There are some remaining issues to be investigated. First, we used the same mid-frequency ranges for /s/ and /ʃ/ sounds, rather than two different frequency ranges as in Shadle *et al.* (2018). In that study, the authors compared /s/ and /ʃ/ production in two individuals who had undergone glossectomy and healthy controls. The spectral peak was located within 2–4 kHz for /ʃ/ sounds. It is unclear whether using a more narrowly defined range for /ʃ/ would improve measurement accuracy and better align with VAS ratings. Nonetheless, in our case, using the same mid-frequency range for /s/ and /ʃ/ is justified because this would allow us to include the misarticulated tokens (e.g., substitution of /ʃ/ with [s]) within our large sample size. Our results also indicate that the spectral peak for [ʃ] in the current data set is well above 4 kHz and, therefore, using a narrower, lower mid-frequency range specifically for /ʃ/ is not indicated.

The discrepancies in methods of measurement also lead to the second limitation of our study. The mean *F_M_* in 3–9 kHz for [s] sound (*M* = 6769 Hz) is much higher than the adolescent's values reported in Koenig *et al.* (2013)—around 4401–4843 Hz in labialized and 5742–6320 Hz in non-labialized context. It is evident from Fig. 1 that the data for /s/ targets is skewed to the right. Some data points were very close to the ceiling of the mid-frequency range, particularly for *F_M_* in 3–7 kHz and *F_M_* in 3–8 kHz. Whether such a result is driven by children's hyper-articulation of [s], vocal-tract size difference or measurement error requires further investigation.

Finally, the stimuli presented in this study include vocalic information (150 ms of the vowel following the fricative), but second formant frequency (F2) at the vowel onset was not measured nor included in the modeling. Although Li (2012) found that onset F2 did not distinguish /s/ and /ʃ/ in English-speaking adults and children aged 2–5 years old, we could not rule out the possibility that onset F2 may contribute to the perception of place of articulation of these two English fricatives in our study, as in Li *et al.* (2011). Further investigation is required to determine the role of onset F2 in the perception of children's fricative when compared to M1 and *F_M_*. For example, fricative measures might be best expressed relative to the resonant properties of the following vowel, as in Winn and Munson (2022).

## 4. Conclusion

Our results provided strong evidence that spectral peak at mid-frequency better predicts VAS ratings of children's /s/ and /ʃ/ sounds than spectral centroid. We also find that for measuring 2.5–3.5-year-old children's sibilants, 3–9 kHz is a more appropriate mid-frequency range than 3–7 kHz, which was used for adolescents and adult speech in previous studies. However, future research is needed to ensure that this wider range is not over-applied. Even in our corpus of young children's fricatives, 5% of tokens had suspicious measures on this broader range. Future study should also examine the utility of *F_M_* and the other alternative measures related to the degree of sibilance and noise source, proposed by Shadle and colleagues, for measuring children's sibilant production, including sibilants that are produced as approximants.

## Supplementary Material

See the supplementary material for the fixed effect statistics of best-fitted models of spectral centroid (M1), spectral peak (*F_M_*) in 3–7 kHz range, *F_M_* in 3–8 kHz range, and *F_M_* in 3–9 kHz range.

## Data Availability

The data that support the findings of this study are available from the corresponding author upon reasonable request.
